# A monkey antigen crossreacting with carcinoembryonic antigen, CEA.

**DOI:** 10.1038/bjc.1976.176

**Published:** 1976-10

**Authors:** E. Engvall, M. Vuento, E. Ruoslahti

## Abstract

**Images:**


*Br. J. Cancer* (1976) **34,** 341

### A MONKEY ANTIGEN CROSSREACTING WITH CARCINOEMBRYONIC ANTIGEN, CEA*

E. ENGVALL,† M. VUENTO AND E. RUOSLAHTI

*From the Department of Immunology, City of Hope National Medical Center, Duarte, California,* 91010 (*present address*) *and the Department of Serology and Bacteriology, University of Helsinki, Finland*

Received 4 April 1976 Accepted 17 June 1976

**Summary.—Normal monkey tissues were found to contain an antigen which crossreacts immunologically with the carcinoembryonic antigen (CEA) of the human digestive tract. The monkey antigen reacted with complete or partial identity to the normal crossreacting antigen (NCA) in humans when tested in immunodiffusion against anti-CEA or anti-NCA. Extracts of monkey tissues inhibited in radioimmunoassays measuring human NCA. It is possible that monkey foetuses and colonic tumours contain CEA.**

THE LACK of an animal model for carcinoembryonic antigen, CEA, (Gold and Freedman, 1965) is a serious drawback in the study of this important tumour antigen. Rat tumours have been found to contain antigens resembling CEA in some of their properties (Stevens *et al.*, 1975; Abeyonis and Milgrom, 1976), but immunological and biochemical evidence that they are related to human CEA is lacking.

We have recently shown that Cynomolgus monkeys immunized with CEA produce antibodies which are more specific for CEA than those produced by rabbits or sheep (Ruoslahti *et al.*, 1976). The monkeys did not react against the determinants which are common to CEA and NCA, the normal crossreacting antigen (von Kleist, Chavanel and Burtin, 1972; Mach and Pusztaszeri, 1972; Darcy, Turberville and James, 1973). Other species invariably form antibodies to NCA in addition to antibodies to CEA when immunized with highly purified CEA. This suggested that the monkeys have themselves a normally occurring antigen similar to NCA. We show here that monkey tissues contain an antigen which is immunologically closely related to human NCA and crossreacts with CEA.

### MATERIALS AND METHODS

*Antigens.*—CEA was purified from liver metastases from colon carcinomas (Coligan *et al.*, 1972; Hammarström *et al.*, 1975). NCA was purified from human spleens. Pooled spleens obtained at autopsy were homogenized in water. Glycoproteins were extracted by 1 M perchloric acid (PCA), dialysed against water, and lyophilized. NCA from spleen PCA extracts was bound to an immunoadsorbent prepared from unabsorbed anti-CEA and eluted with 10 M urea in 25% formic acid (Vuento, Engvall and Ruoslahti, 1976). After dialysis of the eluate, NCA was absorbed to Con-A-Sepharose (Pharmacia Fine Chemicals, Uppsala, Sweden) and eluted with 1 M α-D-manno-pyranoside (Sigma Chemical Co., St. Louis, Mo.). Monkey tissues (*Macaca irus*) were purchased from the National Bacteriological Laboratory, Solna, Sweden. They were extracted with 1 M PCA as above.

*Antisera.*—Antisera against CEA were raised in rabbits (Ruoslahti *et al.*, 1976). If haemagglutinating, the antisera were absorbed with well washed red blood cells. Antiserum against NCA (8G3), raised in a goat, was

* This work was supported by the Finnish Medical Research Council, the Cilag-Chemie Foundation, and the National Cancer Institute (Grant No. CA 16434).
† EMBO Fellowship recipient (ALTF 218-1974).

kindly provided by Dr A. M. Neville, Chester Beatty Research Institute, London.

*Immunological tests.*—Radioimmunoassay of NCA was performed by double antibody technique using either one of two assays. One used CEA as tracer and anti-NCA as antibody. The other used NCA as tracer and anti-CEA as antibody. Iodination with [$^{125}$I]Na (New England Nuclear) was done by means of Chloramine-T. Immunodiffusion plates were from Hyland Laboratories, Costa Mesa, California.

### RESULTS

Immunopurification followed by fractionation on Con A-Sepharose yielded about 1 mg of purified NCA per gram PCA extract of human spleen. This represented an approximate yield of 10% based on the radioimmunoassay value of the initial extract. Goat anti-NCA and rabbit anti-CEA sera bound the same amount (60–80%) of radiolabelled NCA preparations. Upon gel filtration on Sephadex G-200, radiolabelled NCA eluted as a broad peak between human serum albumin and ovalbumin. In immunodiffusion, purified NCA gave a precipitation line with antisera to CEA, showing partial identity with that of CEA (Fig. 1). When tested against anti-NCA, the preparations gave a precipitation line which fused completely with that of CEA (not shown).

PCA extract of monkey spleen or lung reacted in immunodiffusion with rabbit anti-CEA, giving a precipitation line which fused with that of human NCA (Fig. 1). Some antisera revealed a reaction of partial identity between the monkey antigen and human NCA, while other sera did not differentiate between the two antigens.

In radioimmunoassay, monkey spleen extract inhibited the reaction of CEA with anti-NCA and NCA with anti-CEA (Fig. 2). However, the slope of the inhibition curve was less steep than that of the

FIG. 1.—Immunodiffusion. CEA (1 mg/ml), NCA: (1 mg/ml), M = monkey spleen PCA extract (50 mg/ml); tested against anti-CEA.

FIG. 2.—Inhibition of binding of [$^{125}$I] NCA to anti-CEA by purified NCA (●), purified CEA (▲), and PCA extract of human (○), monkey (△), and rat (□) spleen, compared on a weight basis.

FIG. 3.—Gel filtration of monkey spleen extract on a 15 × 110 cm column of Sephadex G-200 in phosphate buffered saline, pH 5·5. ○ : absorbance at 280 nm. ● : inhibition in radioimmunoassay expressed as ng/ml NCA. Arrows indicate the elution volumes of the following substances: CEA, IgG, HSA (human serum albumin), NCA, and OA (ovalbumin).

standard (NCA or CEA) or that of human spleen extract. On a weight basis, monkey spleen extract was approximately 100 times less potent an inhibitor than human spleen extract. No inhibition was obtained with rat spleen extract.

When monkey spleen extract was fractionated on Sephadex G-200, two peaks of activity in radioimmunoassay were found (Fig. 3). One corresponded to an elution volume comparable to that of purified NCA. Complete inhibition curves were not done separately on the two immunoactive peaks, due to the limited amount of material available.

### DISCUSSION

The data presented here demonstrate the presence of NCA in Cynomolgus monkeys. To our knowledge, this is the first time an antigen with a demonstrated relationship to CEA has been found in a non-human species.

The monkey antigen is, like human NCA, present in normal spleen and lung. It reacts strongly with anti-CEA and anti-NCA in immunodiffusion. Surprisingly, it was a rather poor inhibitor in radioimmunoassays. It was recently shown in another system that antigens which were found to be indistinguishable in immunodiffusion behaved differently in radioimmunoassay (Marcus and Zinberg, 1975). Gel filtration of monkey spleen extract gave two peaks of activity in radioimmunoassay. One eluted with an apparent molecular weight similar to that of NCA, the other as a higher molecular weight material. We do not know whether these two peaks are due to two different antigens or aggregation of a single component.

CEA is associated with the membrane of cancerous cells of entodermal origin (Herberman *et al.*, 1975), NCA with the membrane of certain normal leucocytes (Bordes, Knobel and Martin, 1975). The two antigens crossreact immunologically. The basis of this crossreaction is not understood. Our recent data (to be published) indicate that it is not due to similarities in the carbohydrate moieties of the two molecules, in which case the crossreaction could be fortuitous, but to similarities in the protein parts. This suggests that CEA and NCA in humans have a common gene or ancestor gene, and that the monkey equivalent of CEA should be found in monkey foetuses and monkey colonic tumours. Search for CEA in monkeys may therefore lead to establishment of an animal model for CEA.

The authors are greatly indebted to Miss Sirpa Kuisma for excellent technical assistance, to Dr Munro Neville for the anti-NCA serum, and to Drs Svante Stenman and Anders Norling for tissue samples.

### REFERENCES

ABEYOUNIS, C. J. & MILGROM, F. (1976) A Thermostable Antigen Characteristic for Carcinogen-Induced Rat Intestinal Tumours. *J. Immunol.*, **116**, 30.

BORDES, M., KNOBEL, S. & MARTIN, F. (1975) Carcinoembryonic Antigen (CEA) and Related Antigens in Blood Cells and Hematopoietic Tissues. *Eur. J. Cancer*, **11**, 783.

COLIGAN, J. E., LAUTENSCHLEGER, J. T., EGAN, M. L. & TODD, C. W. (1972) Isolation and Characterization of Carcinoembryonic Antigen. *Immunochemistry*, **9**, 377.

DARCY, D. A., TURBERVILLE, C. & JAMES, R. (1973) Immunological Study of Carcinoembryonic Antigen (CEA) and a Related Glycoprotein. *Br. J. Cancer*, **28**, 147.

GOLD, P. & FREEDMAN, S. P. (1965) Specific Carcinoembryonic Antigens of the Human Digestive System. *J. exp. Med.*, **122**, 467.

HAMMARSTRÖM, S., ENGVALL, E., JOHANSSON, B. G., SVENSSON, S., SUNDBLAD, G. & GOLDSTEIN, I. J. (1975) Nature of the Tumor Associated Determinants of Carcinoembryonic Antigen (CEA). *Proc. natn. Acad. Sci. U.S.A.*, **72**, 1528.

HERBERMAN, R. D., AOKI, T., CANNON, G., LIU, M. & STURM, M. (1975) Location by Immunoelectron Microscopy of Carcinoembryonic Antigen on Cultured Adenocarcinoma Cells. *J. natn. Cancer Inst.*, **55**, 797.

MACH, J.-P. & PUSZTASZERI, G. (1972) Carcinoembryonic Antigen (CEA): Demonstration of a Partial Identity Between CEA and a Normal Glycoprotein. *Immunochemistry*, **9**, 1031.

MARCUS, D. M. & ZINBERG, N. (1975) Measurement of Serum Ferritin by Radioimmunoassay: Results in Normal Individuals and Patients with Breast Cancer. *J. natn. Cancer Inst.*, **55**, 791.

RUOSLAHTI, E., ENGVALL, E., VUENTO, M. & WIGZELL, H. (1976) Monkey Antisera with Increased Specificity to Carcinoembryonic Antigen (CEA). *Int. J. Cancer*, **17**, 358.

STEVENS, R. H., ENGLAND, C. W., OSBORNE, J. W., CHENG, H. F. & RICHERSON, H. B. (1975) Oncofetal Protein Accompanying Irradiation-induced Small-bowel Adenocarcinoma in the Rat. *J. natn. Cancer Inst.*, **55**, 1011.

VON KLEIST, S., CHAVANEL, G. & BURTIN, P. (1972) Identification of an Antigen from Normal Human Tissue that Crossreacts with the Carcinoembryonic Antigen. *Proc. natn. Acad. Sci. U.S.A.*, **69**, 2492.

VUENTO, M., ENGVALL, E. & RUOSLAHTI, E. (1976) Purification of the Carcinoembryonic Antigen (CEA) with Immunoadsorbents. In *Scand. J. Immunol.*, suppl. 3, Ed. E. Ruoslahti, p. 83.


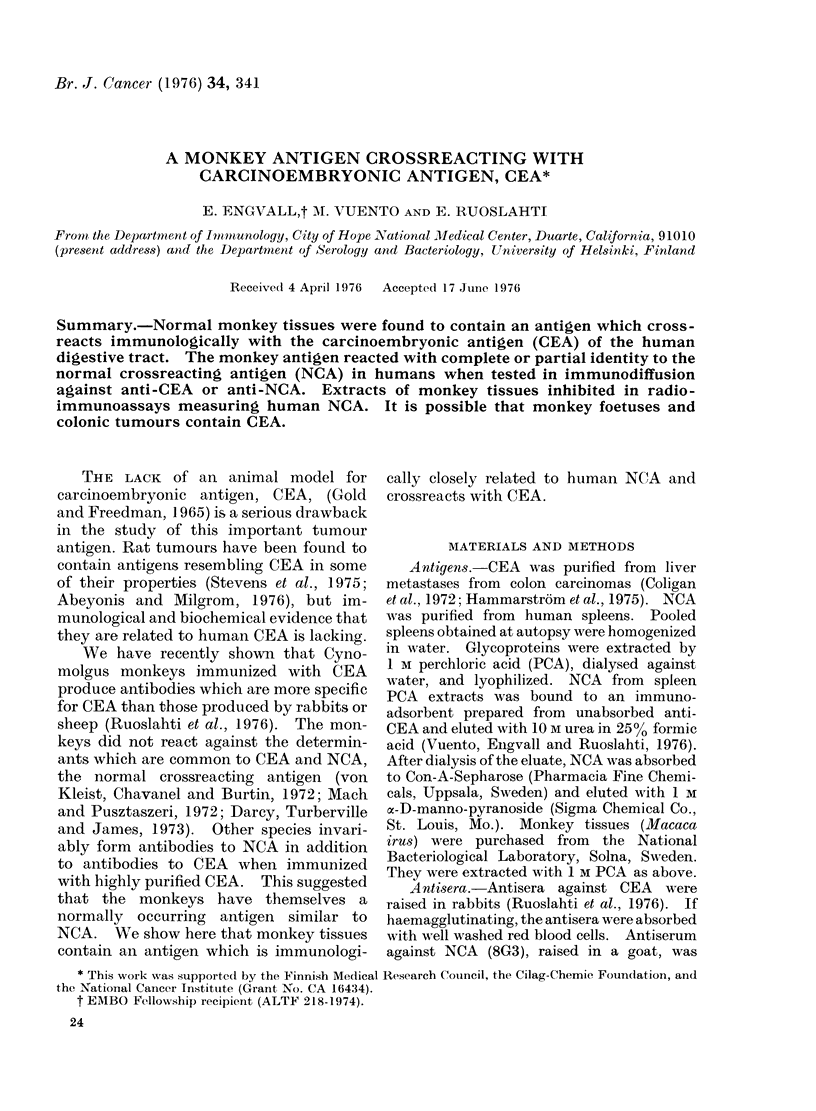

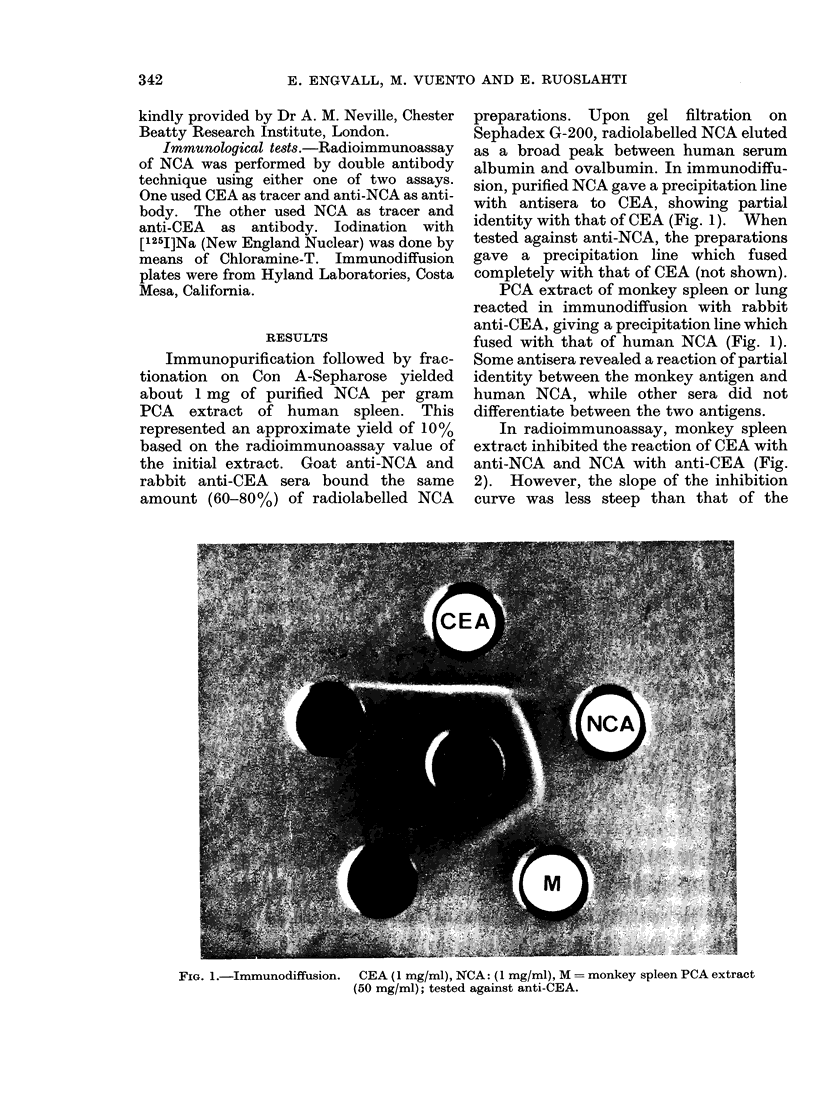

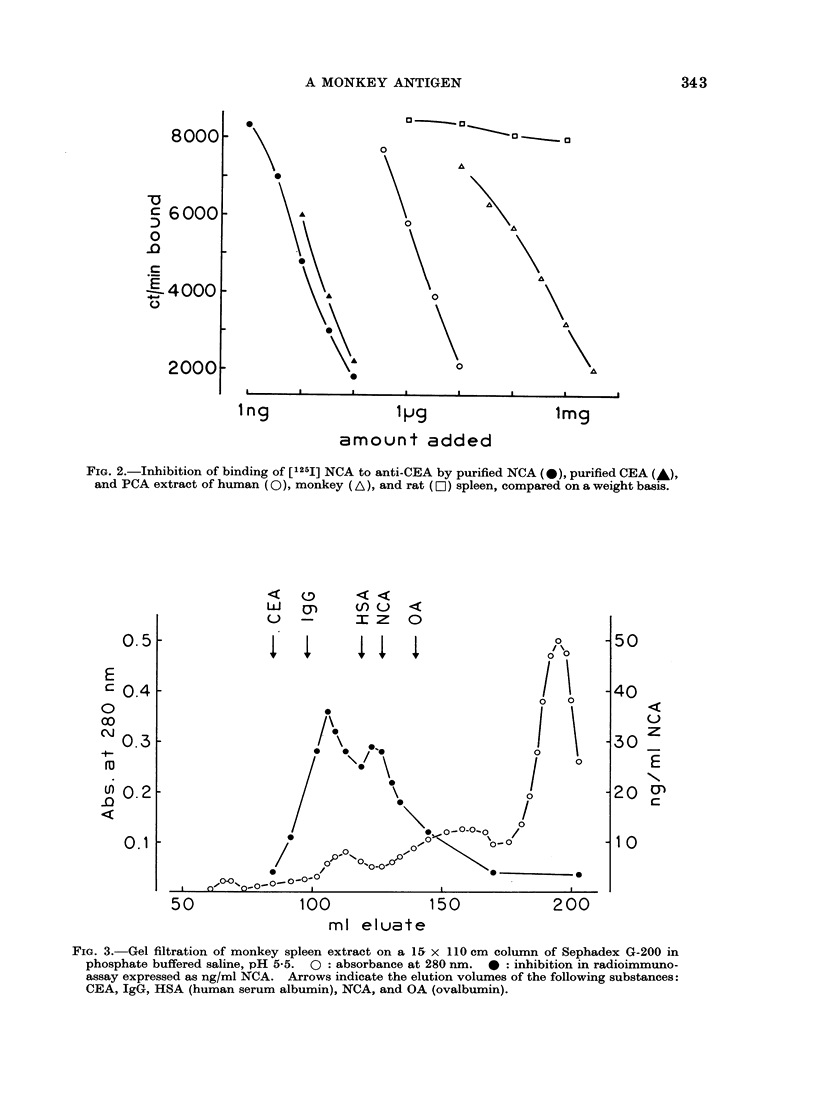

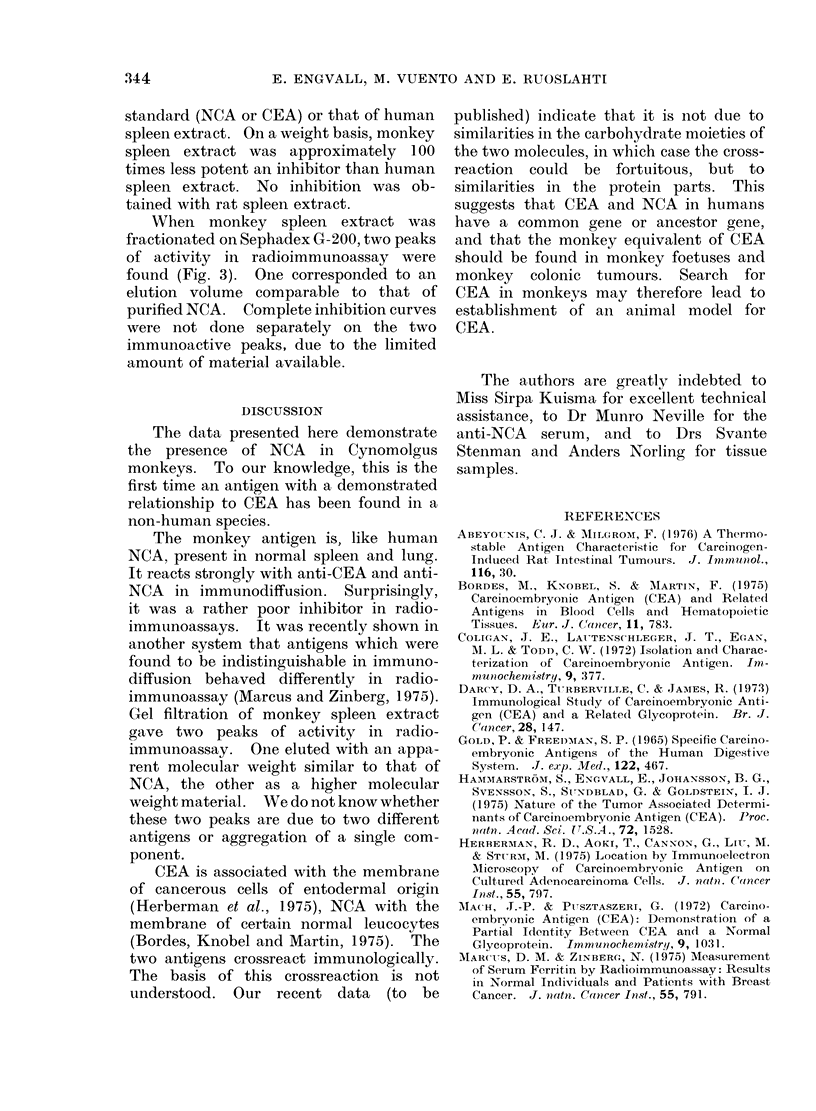

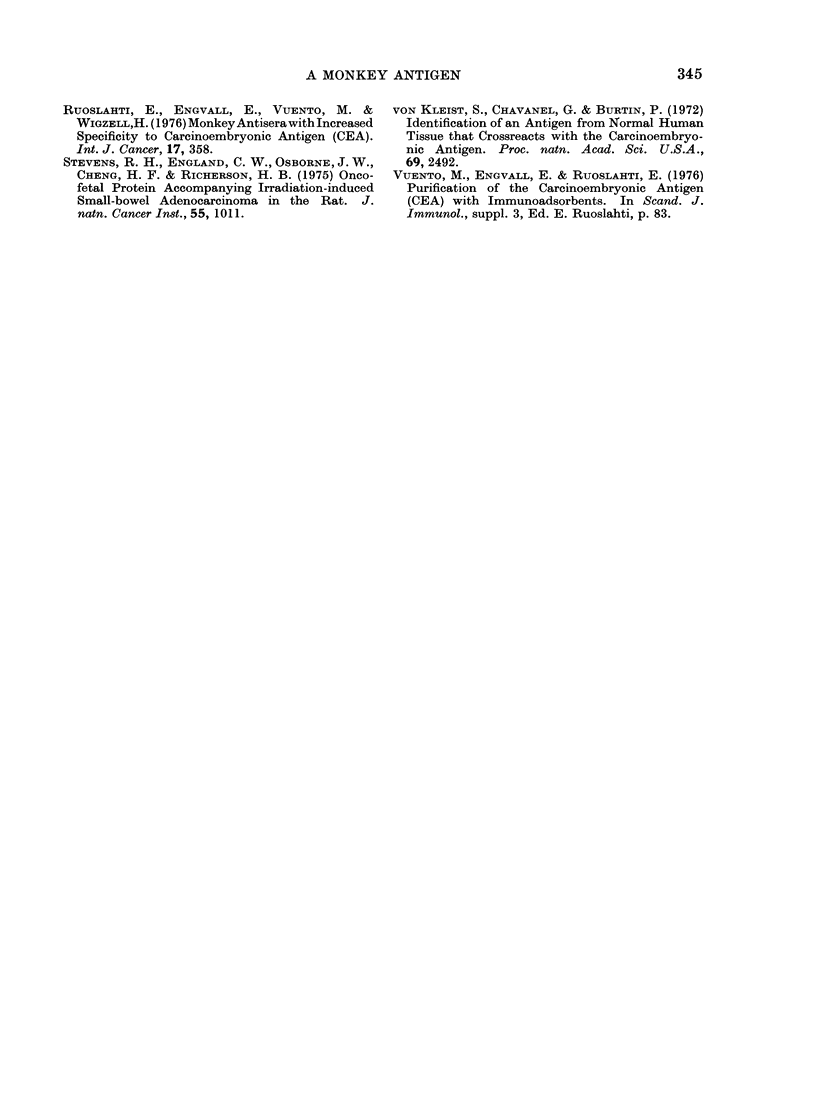


## References

[OCR_00363] Abeyounis C. J., Milgrom F. (1976). A thermostable antigen characteristic for carcinogen-induced rat intestinal tumors.. J Immunol.

[OCR_00375] Coligan J. E., Lautenschleger J. T., Egan M. L., Todd C. W. (1972). Isolation and characterization of carcinoembryonic antigen.. Immunochemistry.

[OCR_00381] Darcy D. A., Turberville C., James R. (1973). Immunological study of carcinoembryonic antigen (CEA) and a related glycoprotein.. Br J Cancer.

[OCR_00387] Gold P., Freedman S. O. (1965). Specific carcinoembryonic antigens of the human digestive system.. J Exp Med.

[OCR_00394] Hammarström S., Engvall E., Johansson B. G., Svensson S., Sundblad G., Goldstein I. J. (1975). Nature of the tumor-associated determinant(s) of carcinoembryonic antigen.. Proc Natl Acad Sci U S A.

[OCR_00399] Herberman R. B., Aoki T., Cannon G., Liu M., Sturm M. (1975). Location by immunoelectron microscopy of carcinoembryonic antigen on cultured adenocarcinoma cells.. J Natl Cancer Inst.

[OCR_00412] Marcus D. M., Zinberg N. (1975). Measurement of serum ferritin by radioimmunoassay: results in normal individuals and patients with breast cancer.. J Natl Cancer Inst.

[OCR_00420] Ruoslahti E., Engvall E., Vuento M., Wigzell H. (1976). Monkey antisera with increased specificity to carcino-embryonic antigen (CEA).. Int J Cancer.

[OCR_00426] Stevens R. H., England C. W., Osborne J. W., Cheng H. F., Richerson H. B. (1975). Oncofetal protein accompanying irradiation-induced small-bowel adenocarcinoma in the rat.. J Natl Cancer Inst.

[OCR_00433] von Kleist S., Chavanel G., Burtin P. (1972). Identification of an antigen from normal human tissue that crossreacts with the carcinoembryonic antigen.. Proc Natl Acad Sci U S A.

